# Unraveling circRNAs and ceRNA regulatory networks underlying sex-specific gametogenesis impairment in triploid Pacific oysters (*Crassostrea gigas*)

**DOI:** 10.1186/s13293-026-00958-x

**Published:** 2026-07-24

**Authors:** Lang Guo, Huihui Wang, Hong Yu, Qi Li

**Affiliations:** 1 Key Laboratory of Mariculture, Ministry of Education, Ocean University of China, Qingdao, 266003 China; 2 Laboratory for Marine Fisheries Science and Food Production Processes, Qingdao Marine Science and Technology Center, Qingdao, 266237 China

**Keywords:** Gametogenesis impairment, Triploid, CircRNA, CeRNA network, *Crassostrea gigas*

## Abstract

**Background:**

In the Pacific oyster *Crassostrea gigas*, triploidization—a well-established technique for generating sterile organisms—provides a promising model to study reproductive dysfunction, as it causes severe and sex-specific impairments in gametogenesis. Specifically, triploid males produce abundant spermatocytes and spermatids but fail to generate mature spermatozoa, while oogenesis in triploid females presents a more complex and heterogeneous picture. The molecular basis for this differential gametogenesis impairment, however, is not fully understood.

**Methods:**

To investigate the roles of circRNA and ceRNA networks in the sex-specific gametogenesis impairment of triploid oysters, gonadal circRNAs were identified and their expression was profiled from whole transcriptome data of diploid and triploid oysters. Differentially expressed circRNAs were then subjected to functional enrichment analysis. Competitive endogenous RNA (ceRNA) networks were constructed from the interactions of differentially expressed RNAs. Key candidate genes implicated in gametogenesis impairment were then identified and characterized through integrated bulk and single-nucleus RNA-seq analyses.

**Results:**

Differential expression analysis revealed substantial alterations in circRNAs expression between diploid and triploid gonads. Functional enrichment of circRNA source genes implicated global metabolic and cell proliferation pathways associated with oogenesis impairment, while it implicated lipid/steroid metabolism, cell proliferation, and apoptosis pathways associated with spermatogenesis disruption in triploid oysters. Sex-specific ceRNA networks were constructed for oogenesis and spermatogenesis, revealing extensive regulatory rewiring in triploid gonads. Analysis of bulk and single-nucleus RNA-seq data identified key candidate genes within these ceRNA networks. Specifically, upregulation of *FoxG1*, *Sox11*, *Cyp2J6*, and *Tra2A* in somatic cells, combined with downregulation of *Uhrf1*, *Ccna2*, *Sox2*, and *Bre1* in germ cells, was implicated in oogenesis impairment of triploids. Similarly, the ceRNA-mediated suppression of *Iqch*, *Ccdc173*, *Srebp1*, and *Gtpbp2* was linked to disrupted spermatogenesis.

**Conclusions:**

These findings highlight that circRNA-mediated ceRNA dysregulation is potentially associated with gametogenesis impairment in triploid oysters, and offer novel insights into post-transcriptional regulatory mechanisms underlying molluscan reproduction.

**Supplementary Information:**

The online version contains supplementary material available at https://doi.org/10.1186/s13293-026-00958-x.

## Abstract (plain language summary)

Owing to their sterile and fast-growing nature, triploid oysters are now a staple of global oyster aquaculture. This impaired reproductive function provides an important model to study reproductive dysfunction. However, the potential mechanisms regulating the differential impairment of gametogenesis in female versus male triploid oysters are not fully understood. This study identified circRNAs and profiled their expression using whole transcriptome data of diploid and triploid oyster gonads. Many differentially expressed circRNAs were identified between diploid and triploid gonads. In females, functional enrichment analysis of differentially expressed circRNA source genes revealed the involvement of global metabolic and cell proliferation pathways in oogenesis impairment in triploid oysters. In males, the analysis revealed the association of lipid/steroid metabolism, cell proliferation, and apoptosis pathways with spermatogenesis disruption. The research constructed sex-specific competitive endogenous RNA (ceRNA) networks. Significant perturbations in the regulatory landscape of triploid gonads were found, which is potentially associated with impairment of gametogenesis. This study further identified key candidate genes within ceRNA networks by using gonadal bulk and single-nucleus RNA-seq data. Detailly, the expression of *FoxG1*, *Sox11*, *Cyp2J6*, and *Tra2A* increased in somatic cells, while expression of *Uhrf1*, *Ccna2*, *Sox2*, and *Bre1* decreased in germ cells. These expression alterations potentially lead to oogenesis impairment of triploids. Meanwhile, the expression of *Iqch*, *Ccdc173*, *Srebp1*, and *Gtpbp2* was suppressed via ceRNA mechanism, which likely contribute to spermatogenesis disruption. Importantly, these genes are highly conserved across species, from humans to oysters, thereby providing new insight with potential implications for understanding reproductive dysfunction broadly.

**Supplementary Information:**

The online version contains supplementary material available at https://doi.org/10.1186/s13293-026-00958-x.

## Highlights

Oogenesis impairment was associated with circRNA-mediated alteration of global metabolic and cell proliferation pathways.

Spermatogenesis disruption was linked to circRNA-mediated dysregulation of lipid/steroid metabolism, cell proliferation, and apoptosis pathways.

The ceRNA networks implicated in sex-specific gametogenesis impairment of triploid oysters were established.

The ceRNA-mediated upregulation of somatic cells-expressed genes (FoxG1, Sox11, Cyp2J6, Tra2A, et al.), and downregulation of germ cells-expressed genes (Uhrf1, Ccna2, Sox2, Bre1, et al.) potentially triggered impaired oogenesis.

Suppression of Iqch, Ccdc173, Srebp1, Gtpbp2, et al. by ceRNA mechanism likely result in disrupted spermatogenesis.

**Supplementary Information:**

The online version contains supplementary material available at https://doi.org/10.1186/s13293-026-00958-x.

## Introduction

Gametogenesis is a critical phase of sexual reproduction, wherein germ cells undergo a series of complex developmental processes to ultimately form mature gametes—spermatozoa and oocytes. Researchers have invested considerable effort in elucidating the mechanisms of gametogenesis using diverse model organisms [1, 2]. Because gametogenesis requires precise coordination of chromosome behavior, cell-cycle progression, and gene expression [3], perturbations in ploidy status often result in profound reproductive defects. Triploidization has emerged as a reliable method for producing sterile organisms and is widely applied across species. Normally, reproduction of natural diploids consumes most of an organism’s energy to produce gametes during the breeding season. In triploids, however, this energy is redirected from reproductive efforts to somatic growth, offering substantial economic advantages [4]. At the same time, triploid organisms provide unique natural models for investigating the molecular basis of gametogenesis impairment.

The Pacific oyster (*Crassostrea gigas*) is a representative marine bivalve with great value in global aquaculture and in the marine ecosystem. Triploid oysters have become an essential component of the global oyster aquaculture industry due to the commercial advantages derived from their impaired reproductive potential [5]. Nevertheless, the effects of triploidy on gametogenesis are sex-specific. Triploid male oysters exhibit numerous primary spermatocytes and spermatids, but few mature spermatozoa. In addition, oogenesis in triploid females is more complex and variable. Previous studies have categorized them into two groups: female α, which possess a large number of oocytes, and female β, which have few or no oocytes [6]. These strikingly divergent reproductive phenotypes highlight the complex and sex-dependent consequences of triploidy on gametogenesis. Accordingly, triploid oysters have intrigued biologists, establishing them as important models for studying gametogenesis impairment and reproductive dysfunction. However, despite extensive phenotypic and cytological characterization, the regulatory mechanisms underlying the impaired gametogenesis in triploid oysters remain largely unresolved.

To investigate this puzzle, previous studies have employed a suite of omics techniques and functional characterization approaches, focusing mainly on elucidating the potential functions of key genes and their regulatory mechanisms [7–14]. Nevertheless, gene expression regulation constitutes a complex interplay of intricately controlled events [15, 16], in which post-transcriptional regulation plays a central role, and non-coding RNAs (ncRNAs) have been implicated as key regulators [17]. Accumulating evidence underscores their pervasive involvement at multiple regulatory levels, demonstrating in particular that they are essential regulators of animal gametogenesis and gonadal development. For instance, human studies have shown that several miRNA clusters, including miR-106b-25 and miR-17-92, as well as miR-484, play significant roles in spermatogenesis [18, 19]. In *Drosophila melanogaster*, Lsd1-interacting lncRNAs and hairpin RNAs have been identified as regulators of oogenesis and spermatogenesis, respectively [20, 21]. Recent studies across diverse taxa have increasingly highlighted the importance of circRNAs in reproductive development, including spermatogenesis, oogenesis, and follicular growth [22–26].

A core mechanism of ncRNA function is the competing endogenous RNA (ceRNA) network, which arises through crosstalk among mRNAs, lncRNAs, and circRNAs that competitively bind shared miRNAs. It is now well-established that ceRNA networks are ubiquitous in animals and regulate diverse biological processes. Notably, their regulatory roles have been demonstrated in both vertebrates and invertebrates [23–25, 27–29]. Given that triploidy disrupts gene dosage, meiotic progression, and transcriptional balance, ceRNA-mediated regulation may represent a particularly important mechanism contributing to gametogenesis impairment. However, in molluscs, and particularly in the context of disrupted gametogenesis in triploid oysters, the composition, regulatory relationships, and functional relevance of circRNA-centered ceRNA networks remain largely unexplored.

To this end, circRNAs were systematically identified and their expression profiled using whole transcriptome data from diploid and triploid oyster gonads, followed by functional enrichment analysis of differentially expressed circRNAs. CeRNA networks were constructed by integrating interactions among differentially expressed RNAs. By combining bulk RNA-seq and single-nucleus RNA-seq analyses, key candidate genes associated with gametogenesis impairment were further identified and characterized. This study aims to elucidate how circRNAs and ceRNA networks are associated with impaired gametogenesis in triploid oysters, thereby shedding light on the molecular basis of reproductive dysfunction in molluscs.

## Materials and methods

### Identification of circRNAs in diploid and triploid gonads

All gonad specimens used for sequencing were sampled from two‑year‑old diploid and triploid oysters (three biological replicates) collected in Rongcheng, China. Gonads from the same individuals were used to construct both strand‑specific cDNA libraries and small RNA libraries. These libraries were sequenced in our previous study, and the raw datasets have been deposited in NCBI under BioProject PRJNA690125 (strand‑specific cDNA) and PRJNA791305 (small RNA) [9, 10]. For this study, we downloaded these publicly available datasets from NCBI for re‑analysis. Accession numbers of all sequencing datasets are listed in Supplementary Table S1.

To identify circRNAs, raw reads from strand-specific cDNA sequencing were trimmed and filtered first using fastp (version 0.18.0) [30]. Specifically, reads containing adapters, > 10% unknown nucleotides, or > 50% low-quality bases (Q-value ≤ 20) were removed before downstream analysis to ensure data integrity. Bowtie2 (version 2.2.8) [31] was used to map reads against the ribosome RNA (rRNA) reference database from NCBI GenBank (https://www.ncbi.nlm.nih.gov/genbank/). The rRNA mapped reads were then removed. Subsequently, the rRNA removed reads of each sample were mapped to a *C*. *gigas* reference genome by HISAT2 (version 2.1.1) [32]. The reads that could be mapped to the genomes were discarded, and the unmapped reads were then collected for circRNA identification. 20 mers from both ends of the unmapped reads were extracted and aligned to the reference genome to find unique anchor positions within splice site. Anchor reads that aligned in the reversed orientation (head to tail) indicated circRNA splicing and then were subjected to CIRIquant (version 1.1.2) [33] to identify circRNAs. A candidate circRNA was called if it was supported by at least two unique back spliced reads at least in one sample. For each identified circRNA, we extracted the genomic coordinates of its back-spliced junction (BSJ) and intersected them with the reference genome annotation using custom scripts. Based on the positional relationship between the BSJ and annotated gene models, circRNAs were classified into six categories: antisense, one_exon, intronic, exon_intron, annot_exons, and intergenic. LncRNAs and miRNAs were identified following the pipelines previously described in [9, 12].

### Differential expression analysis between diploid and triploid gonads

The edgeR package (version 3.12.1) [34] was used to detect differentially expressed genes (DEGs), miRNAs (DEMis), circRNAs (DECs), and lncRNAs (DELs) between diploid and triploid gonads, separately for each sex, using the exact test. Significant DEGs, DECs, and DELs were defined by an absolute fold change ≥ 2 and a false discovery rate (FDR) < 0.05, whereas DEMis were identified with an absolute fold change ≥ 2 and *p* < 0.05.

### Construction of ceRNA network

The ceRNA network was constructed based on ceRNA theory [35]. To enable cross‑sample comparison, expression was quantified using library‑size‑normalized metrics: TPM (transcripts per million) for mRNAs and lncRNAs, CPM (counts per million) for miRNAs, and RPM (back‑spliced reads per million) for circRNAs. All three metrics account for sequencing depth, making them directly comparable across samples within each data type. Briefly, expression correlation between mRNA**–**miRNA, lncRNA**–**miRNA and circRNA**–**miRNA was evaluated using the Spearman rank correlation coefficient (SCC). Pairs with SCC < -0.7 were selected as negatively co-expressed pairs, and all RNAs in these pairs were differentially expressed. Expression correlation between ceRNAs was evaluated using the Pearson correlation coefficient (PCC). Pairs with PCC > 0.9 were selected as co-expressed ceRNA pairs. Hypergeometric cumulative distribution function test was used to test whether the common miRNA sponges between ceRNAs were significant (*p* < 0.05). Correlation analyses and hypergeometric cumulative distribution function test were performed using R psych package (version 1.8.4).

### Functional enrichment analysis

We performed Gene Ontology (GO) and Kyoto Encyclopedia of Genes and Genomes (KEGG) enrichment analyses on differentially expressed RNAs. For circRNA analysis, the input was the set of source genes of DECs. For ceRNA network analysis, the input comprised the differentially expressed protein‑coding genes (mRNAs), together with the source genes of circRNAs and the target genes of lncRNAs and miRNAs, all derived from the ceRNA interaction network.

GO enrichment analysis was performed using the GO.db annotation package (version 3.14.0) with default parameters. KEGG pathway enrichment analysis was conducted using the KEGG database (Release 101) with default parameters. All enrichment analyses were performed in the R environment. GO terms and KEGG pathways meeting the threshold of FDR‑adjusted *p* < 0.05 were defined as significantly enriched terms and pathways.

### Spatiotemporal gene expression analysis based on transcriptomic data

Bulk RNA-seq datasets for diploid and triploid male gonads of *C. gigas* at the mature stage (s3) were obtained from the NCBI Sequence Read Archive (SRA) under accession number PRJNA690125 [9]. In addition, this study utilized bulk RNA-seq data (unpublished) generated from diploid and triploid male gonads at the proliferating stage (s1). These data were processed following the transcriptomic pipeline described in [9]. Briefly, raw reads were trimmed and filtered with fastp (version 0.18.0) [30], followed by rRNA removal using Bowtie2 (version 2.2.8) [31]. The rRNA‑depleted reads were then aligned to the reference genome with HISAT2 (version 2.1.1) [32]. Differential expression analysis was performed by DESeq2 [36].

Single-nucleus RNA-seq data of diploid and impaired triploid female gonads in *C. gigas* were retrieved from PRJNA1244049 under accession number SRR35841448, and data were processed using the same pipeline described in [13]. Briefly, after quality control of raw data and alignment to the *C*. *gigas* reference genome, gene expression counts were generated using CellRanger (version 7.1.0). Seurat (version 4.4.0) was used for cell clustering analysis. Nonlinear dimensionality reduction methods (UMAP) were used to visualize and investigate the clusters [37].

### Quantitative real-time PCR validation

Quantitative PCR (qPCR) analysis was performed to validate the expression levels of mRNAs and ncRNAs. Totally, twelve mRNAs, five circRNAs, four lncRNAs, and three miRNAs within the two ceRNA subnetworks were selected. To analyze the expression of mRNAs, circRNAs, and lncRNAs, total RNA was first reverse-transcribed into cDNA using the Evo M-MLV RT Mix Kit (Accurate Biotechnology, China). The qPCR reactions were performed using the SYBR Green Premix Pro Taq HS qPCR Kit (Accurate Biotechnology, China) on a LightCycler^®^ 480 real-time PCR system (Roche, Switzerland). Five candidate reference genes (EF‑1α, actin, GAPDH, 18 S rRNA, and 28 S rRNA) were evaluated for expression stability using geNorm [38] and NormFinder [39]. EF‑1α was identified as the most stable gene and selected as the sole reference gene for RT‑qPCR normalization. To analyze miRNA expression, first-strand cDNA was synthesized from total RNA using a miRNA 1st Strand cDNA Synthesis Kit (by stem-loop) (Vazyme, China). Quantitative PCR (qPCR) of miRNAs was then performed on a LightCycler^®^ 480 system (Roche, Switzerland) using the miRNA Universal SYBR qPCR Master Mix (Vazyme, China). U6 snRNA served as the internal control.

All RT‑qPCR procedures followed the MIQE guidelines [40]. Sequences of all primers designed for qRT-PCR were provided in Supplementary Table S2. Each assay was performed in three biological replicates, and the relative expression was calculated using the 2^−ΔΔCt^ method, with data presented as the mean ± SEM.

## Results

### Identification of circRNAs in oyster gonads

**Fig. 1:**
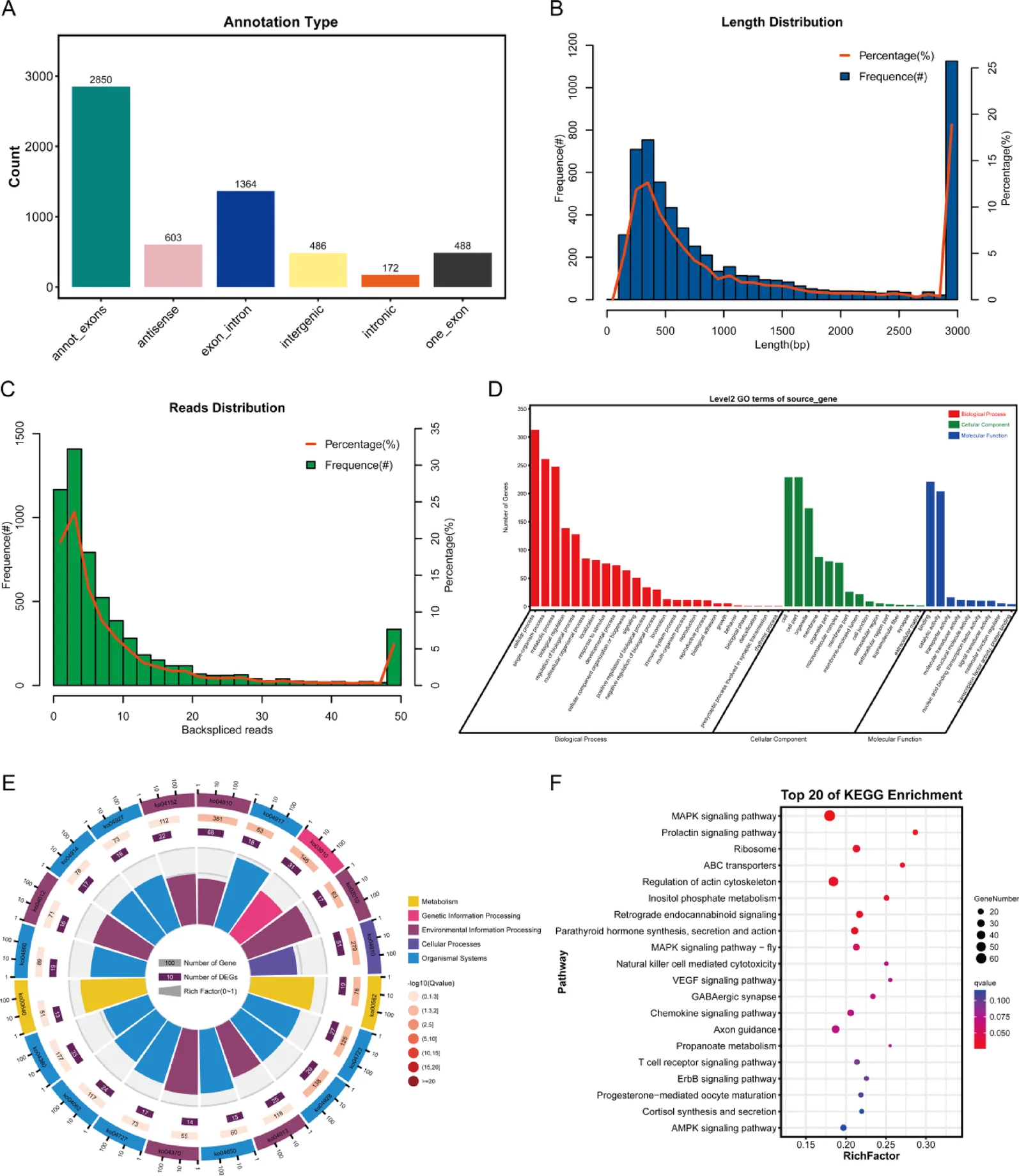
Statistical analysis of gonadal circRNAs and functional enrichment analysis. **A**, Annotation type of circRNAs. **B**, Length distribution of circRNAs. **C**, Reads distribution of circRNAs. **D**, GO enrichment analysis of circRNA source genes. **E**-**F**, KEGG enrichment analysis of circRNA source genes

Fifteen strand‑specific cDNA libraries from gonads of diploid and triploid oysters at the maturation stage, generated in our previous study, were downloaded and re‑analyzed in the present study. After rRNA removal, 82.86%–88.37% of the clean reads were successfully mapped to the *C*. *gigas* reference genome (Supplementary Table S3). A total of 5963 circRNAs were annotated from the fifteen gonadal libraries. Genomic annotation revealed that circRNAs predominantly originated from annotated exons (2850), followed by exon_intron regions (1364) and antisense transcripts (603), with smaller contributions from intergenic (486), one_exon (488) and intronic (172) regions (Fig. 1A). Size distribution analysis revealed that the length of the 5963 circRNAs showed a broad range from 100 to 3000 bp, with a predominant peak in the 300–600 bp range (Fig. 1B). The abundance distribution of back-spliced junction reads revealed a characteristic pattern where the vast majority of identified circRNAs were supported by a low number of reads, marked by a sharp peak at the lowest bin (0–10 reads) and a rapid decline as the read count increased (Fig. 1C).

To further elucidate the potential functions of gonadal circRNAs, GO and KEGG enrichment analyses were performed to the source genes of gonad-expressed circRNA. Results of GO enrichment analysis demonstrated that source genes were mainly enriched in terms such as “cellular process”, “single-organism process”, and “metabolic process” in Biological Process category, “cell”, “cell part” and “organelle” in Cellular component, “binding” and “catalytic activity” in Molecular Function (Fig. 1D). KEGG enrichment analysis revealed that source genes of gonad‑expressed circRNAs were significantly enriched in pathways including the “MAPK signaling pathway”, “Prolactin signaling pathway”, “Ribosome”, and “Regulation of actin cytoskeleton” (Fig. 1E, F).

### Identification and functional analysis of differentially expressed circRNAs

**Fig. 2:**
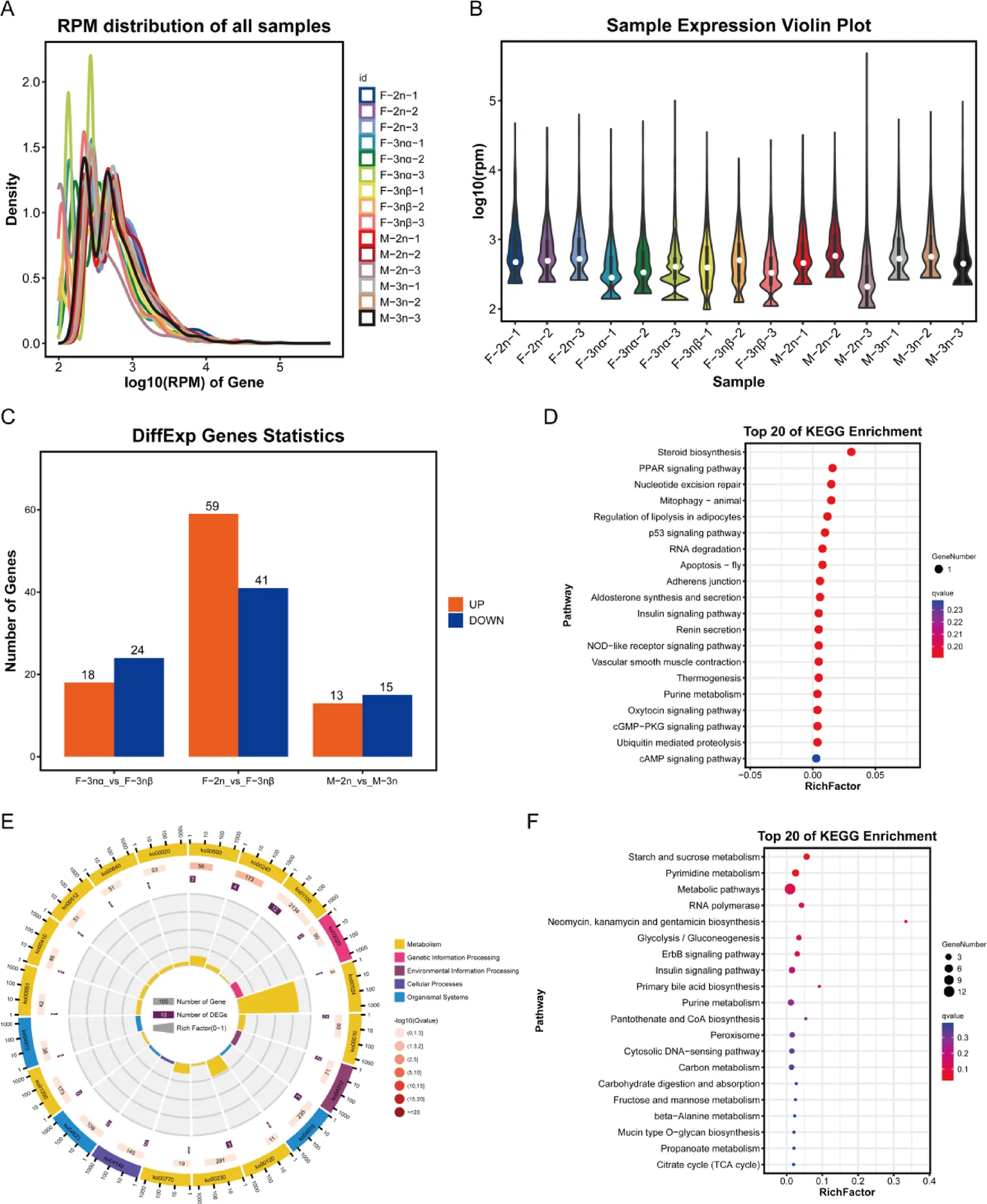
Expression profiles of circRNAs and functional enrichment analysis. **A**, RPM distribution of circRNAs from all samples. **B**, Violin plots of circRNAs expression from all samples. **C**, Number of DECs between diploid and triploid gonads. **D**, KEGG enrichment analysis of source genes of DECs between M-2n and M-3n gonads. E-F, KEGG enrichment analysis of source genes of DECs between F-2n and F-3nβ gonads

To characterize differentially expressed circRNAs between diploid and triploid gonads, we analyzed their expression levels (Fig. 2A, B). Specifically, there were 100 differentially expressed circRNAs (DECs)—59 up-regulated and 41 down-regulated—between diploid female (F-2n) gonads and imp0oaired triploid female (F-3nβ) gonads, of which 95 mapped to annotated source genes. In addition, 44 DECs (39 DECs mapped to annotated source genes) were identified between developed triploid female gonads (F-3nα) and impaired triploid female gonads (F-3nβ). In males, 28 DECs (24 DECs mapped to annotated source genes) were identified between diploid (M-2n) and triploid (M-3n) gonads, with 13 upregulated and 15 downregulated (Fig. 2C).

To investigate the potential roles of these DECs, functional enrichment analyses were performed on their source genes. KEGG enrichment analysis of the comparison between diploid and triploid male gonads revealed enrichment of source genes in pathways including “Steroid biosynthesis”, “PPAR signaling pathway”, “Nucleotide excision repair”, “Regulation of lipolysis in adipocytes”, “RNA degradation”, and “p53 signaling pathway” (Fig. 2D). Meanwhile, source genes of DECs between F-2n and F-3nβ gonads were mainly enriched in metabolism-related pathways, including “Starch and sucrose metabolism”, “Pyrimidine metabolism”, and “Metabolic pathways” (Fig. 2E, F).

### Construction of ceRNA regulatory networks

**Fig. 3:**
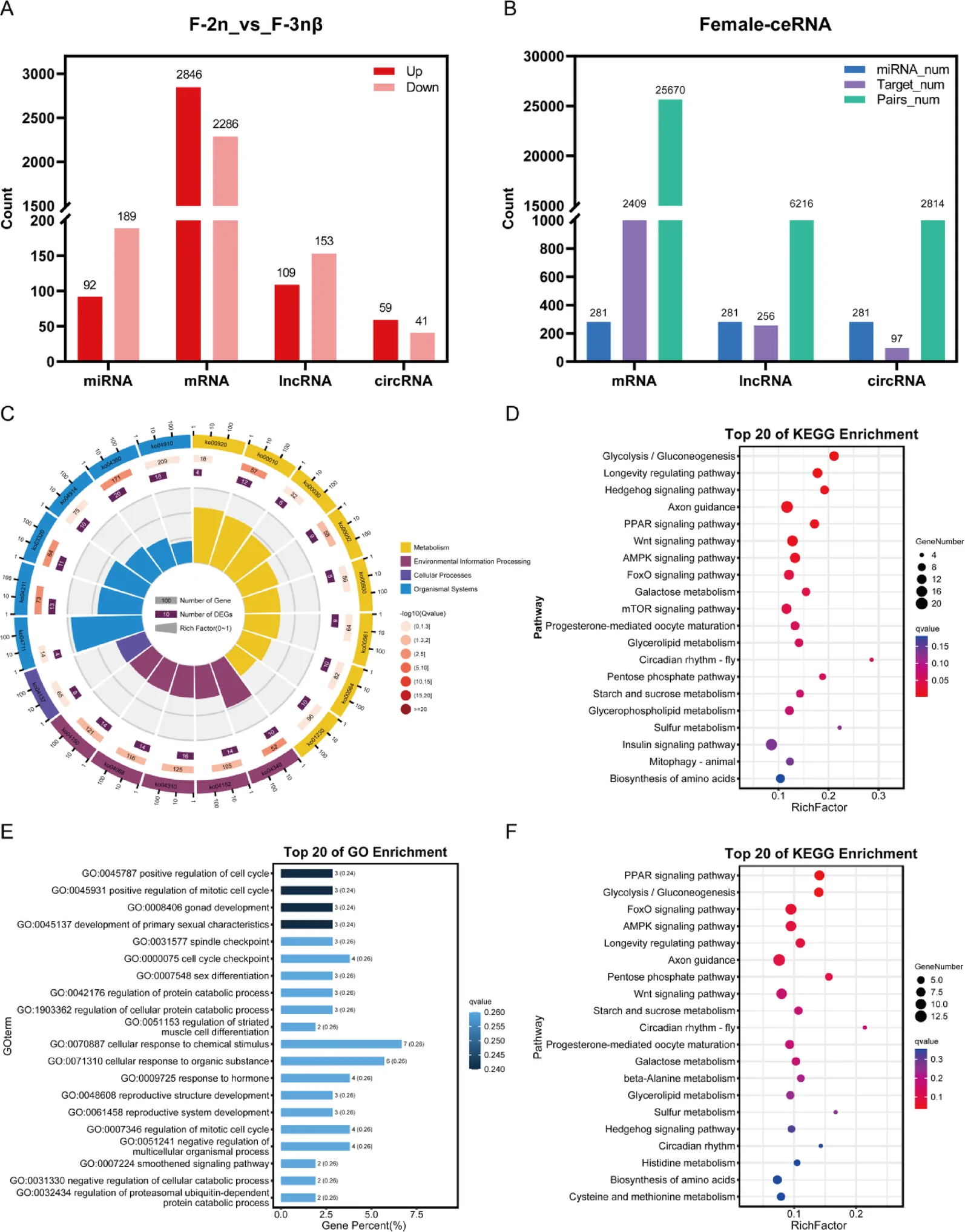
Statistics of differentially expressed RNAs between F-2n and F-3nβ gonads and their ceRNA network. **A**, Number of differentially expressed RNAs between F-2n and F-3nβ gonads. **B**, Components of the F-2n_vs_F-3nβ ceRNA network. **C**-**D**, KEGG enrichment analysis of mRNA in the F-2n_vs_F-3nβ ceRNA network. **E**-**F**, GO and KEGG enrichment analysis of circRNA source genes in the F-2n_vs_F-3nβ ceRNA network

To investigate interactions between differentially expressed protein‑coding and non‑coding RNAs, we integrated DEGs, DELs, DECs, and DEMis (Figs. 3A and 5A) using lncRNA/circRNA–miRNA and miRNA–mRNA interactions for each comparison group. When comparing diploid female gonads with impaired triploid female gonads (F-2n_vs_F-3nβ), we integrated 2409 DEGs, 256 DELs, 97 DECs and 281 DEMis into a ceRNA network (Fig. 3B). Likewise, the comparison between diploid and triploid male gonads (M‑2n_vs_M‑3n) yielded a ceRNA network comprising 267 DEGs, 112 DELs, 26 DECs, and 198 DEMis (Fig. 5B).

To explore the potential roles of the ceRNA networks, we performed enrichment analyses on the protein-coding genes and source/target genes of ncRNAs within the networks. Protein‑coding genes in the F‑2n_vs_F‑3nβ ceRNA network were significantly enriched in numerous KEGG pathways—metabolic pathways including “Glycolysis/Gluconeogenesis,” “Galactose metabolism,” “Glycerolipid metabolism,” “Pentose phosphate pathway,” “Starch and sucrose metabolism,” and “Glycerophospholipid metabolism,” as well as signaling pathways such as “Hedgehog signaling pathway,” “Wnt signaling pathway,” “AMPK signaling pathway,” “Insulin signaling pathway,” “FoxO signaling pathway,” and “PPAR signaling pathway,” (Fig. 3C, D). Meanwhile, source genes of DECs within this ceRNA network were enriched in similar pathways. Besides, these genes were also involved in biological processes such as “positive regulation of cell cycle”, “positive regulation of mitotic cell cycle”, and “cell cycle checkpoint” (Fig. 3E, F).

**Fig. 4:**
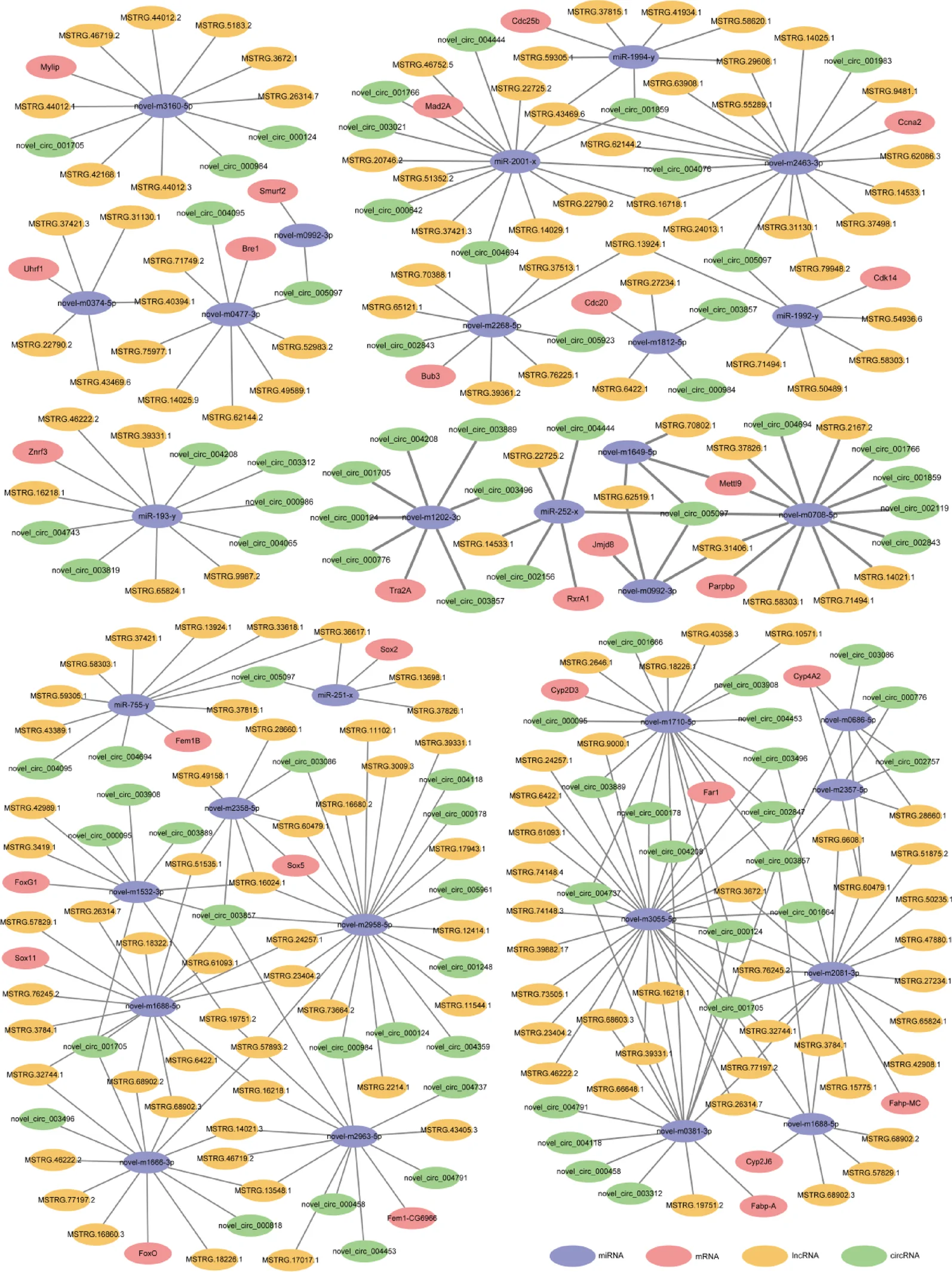
CeRNA subnetwork associated with oogenesis impairment, featuring key candidate genes

**Fig. 5:**
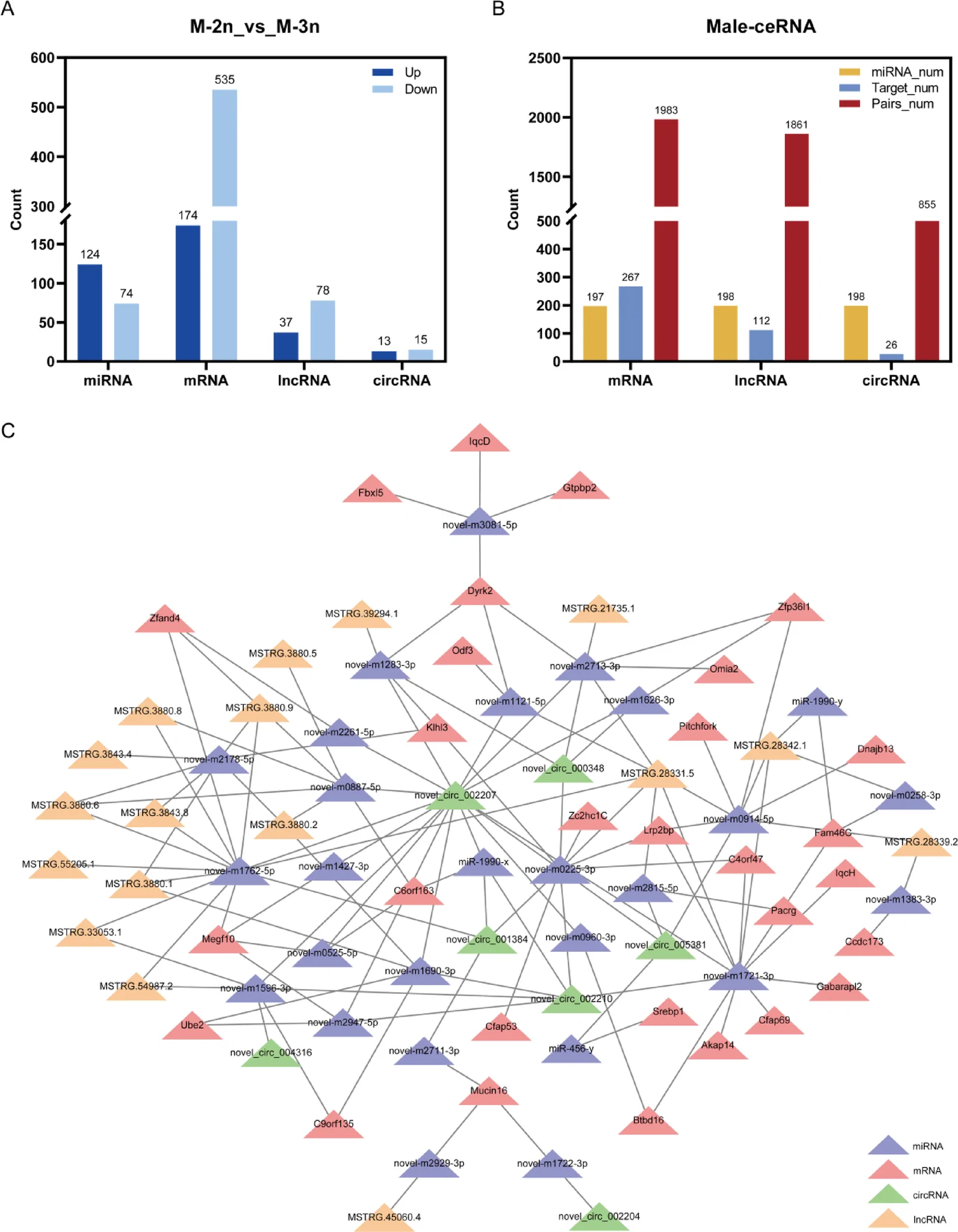
Statistics of differentially expressed RNAs between M-2n_vs_M-3n gonads and their ceRNA network. **A**, Number of differentially expressed RNAs between M-2n_vs_M-3n gonads. **B**, Components of the M-2n_vs_M-3n ceRNA network. **C**, CeRNA subnetwork associated with spermatogenesis disruption, featuring key candidate genes

**Fig. 6:**
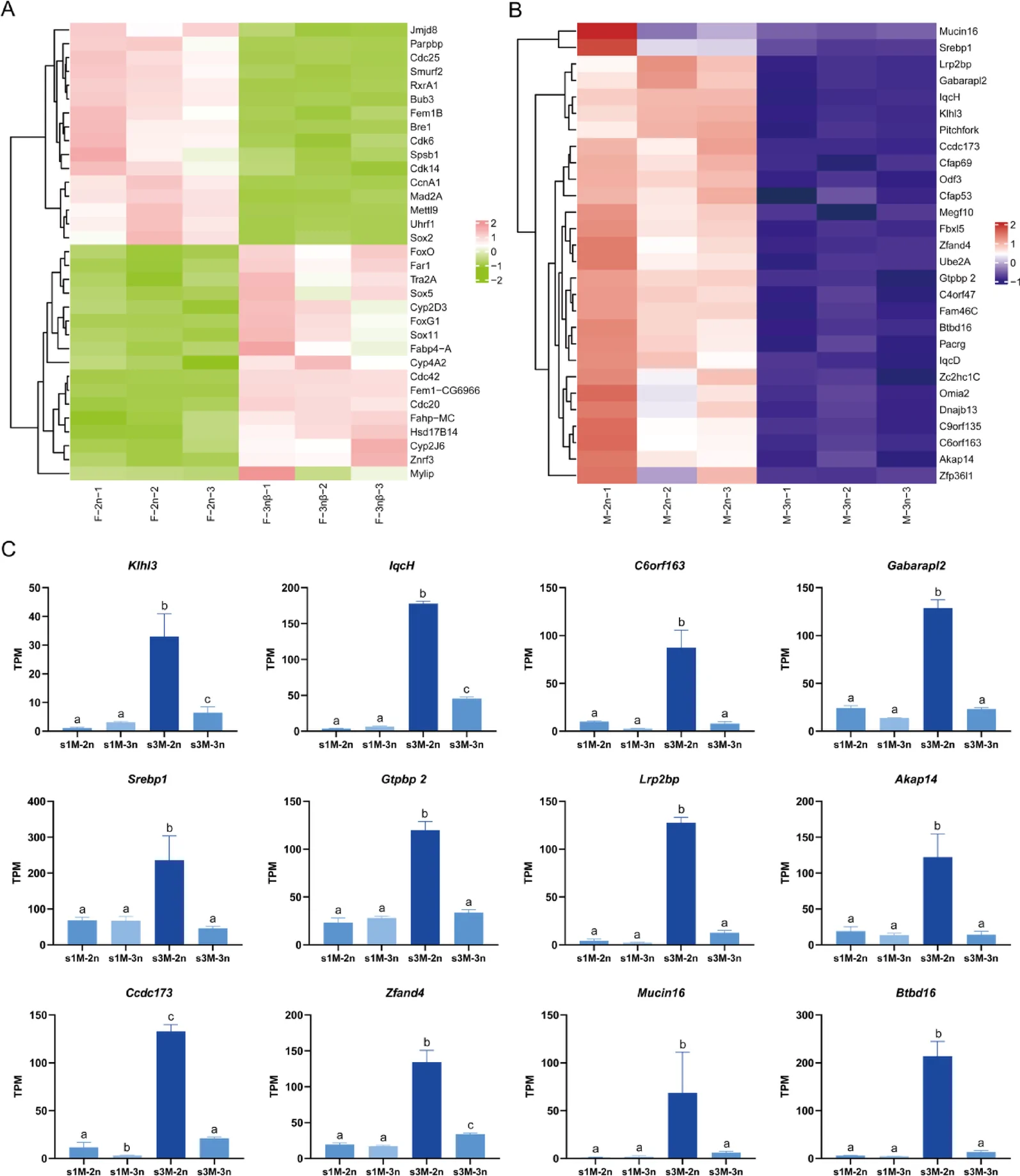
Expression profiles of candidate genes within ceRNA network. **A**-**B**, Heat map of candidate genes in ceRNA subnetworks associated with oogenesis impairment and spermatogenesis disruption. **C**, Expression patterns of candidate genes in ceRNA subnetwork associated with spermatogenesis disruption based on RNA-seq data. Data are presented as mean ± SEM from three biological replicates. Significant differences between groups are indicated by different letters (*p* < 0.05)

To further elucidate the functional roles of the F‑2n_vs_F‑3nβ and M‑2n_vs_M‑3n ceRNA networks, we examined the expression patterns of several key candidate genes potentially involved in gametogenesis. Specifically, we identified several genes highly expressed in diploid female gonads, including *Sox2*, *Mettl9*, *Far1*, *Fem1B*, *Far1*, and *Cdc42*, among others (Fig. 6A). We also found candidate genes with higher expression in impaired triploid female gonads: *FoxO*, *Sox5*, *Fabp4-A*, *Cyp4A2*, *Cyp2J6*, *Mylip*, and others (Fig. 6A). In males, several candidate genes—including *Mucin16*, *Lrp2bp*, *Cfap69*, *IqcH*, *Btbd16*, *Zfp36l1*, among others—showed higher expression in diploid male gonads than in triploid male gonads (Fig. 6B). Subsequently, leveraging these key candidate genes, we derived ceRNA subnetworks associated with oogenesis impairment from the F-2n_vs_F-3nβ ceRNA network (Fig. 4), and a ceRNA subnetwork that was implicated in spermatogenesis disruption from the M-2n_vs_M-3n ceRNA network (Fig. 5C).

### Expression profiles of key candidate genes in diploid and triploid gonads

To explore the potential functions of the candidate genes, we comprehensively examined their expression profiles in diploid and triploid gonads. We analyzed bulk RNA‑seq expression profiles of candidate genes from the M‑2n_vs_M‑3n ceRNA subnetwork in diploid and triploid male gonads across two developmental stages, revealing distinct expression patterns between ploidy and stage groups. Overall, these genes displayed significantly lower expression levels in triploid male gonads at the mature stage, a level similar to that in diploid and triploid gonads at the proliferating stage. Notably, compared to male gonads at the proliferating stage, the expression of *Klhl3*, *IqcH* and *Zfand4* was higher in triploid male gonads at the mature stage. Conversely, *Ccdc173* showed significantly higher expression in diploid male gonads than in triploid ones at both stages (Fig. 6C).

**Fig. 7:**
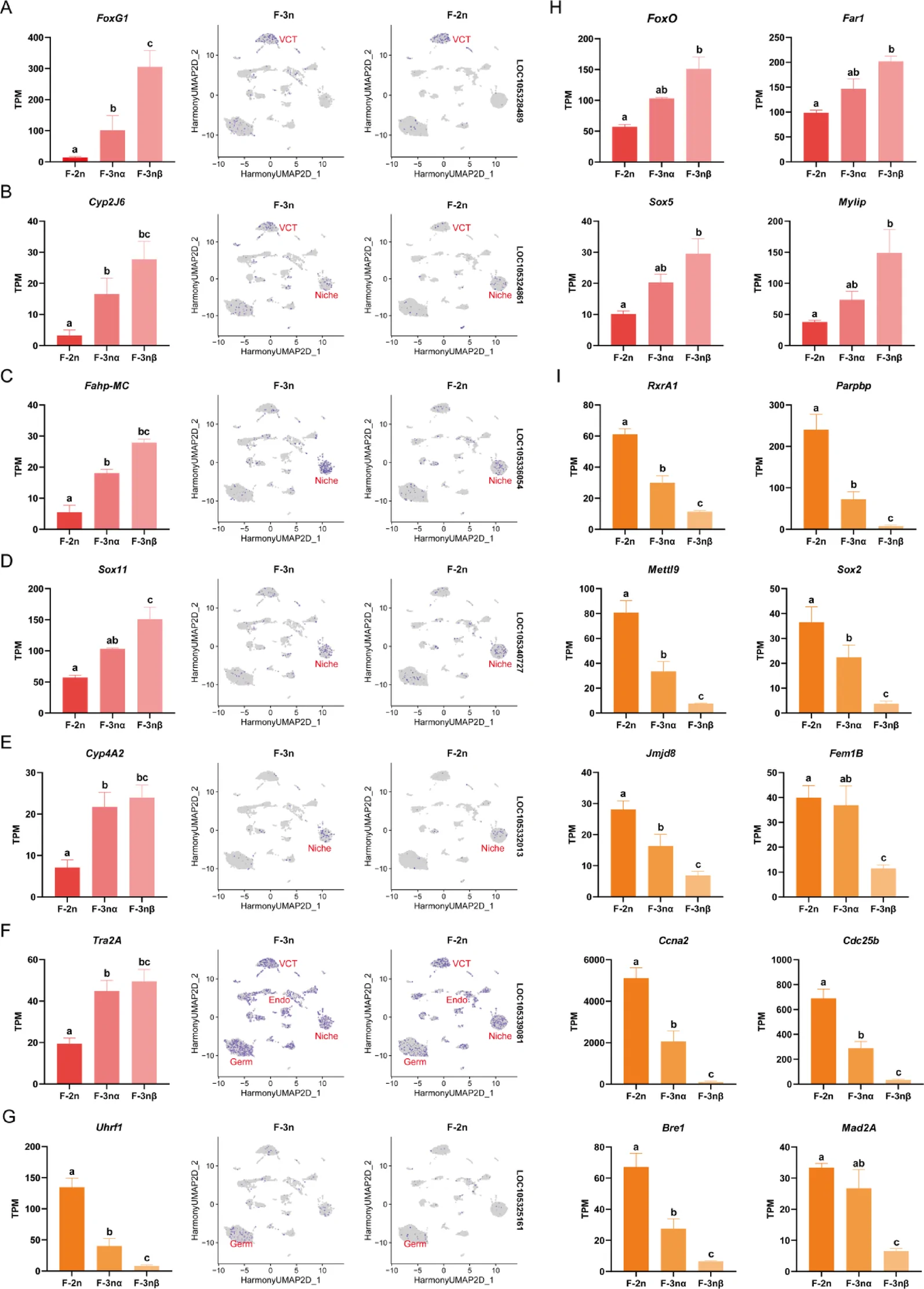
Expression profiles of candidate genes in ceRNA subnetwork associated with oogenesis impairment. **A**-**G**, Expression patterns of candidate genes based on bulk RNA-seq (bar charts on the left) and single-nucleus RNA-seq (UMAP plots on the right) data. **H**-**I**, Expression patterns of candidate genes based on bulk RNA-seq data. Data from bulk RNA-seq data are presented as mean ± SEM from three biological replicates. Significant differences between groups are indicated by different letters (*p* < 0.05). UMAP plots show expression distribution of candidate genes in female gonadal cells

For key candidate genes from the F-2n_vs_F-3nβ ceRNA subnetwork, we performed an integrated analyses on bulk RNA-seq and single-nucleus RNA-seq datasets from diploid gonads and impaired triploid female gonads. Bulk RNA-seq analysis revealed distinct expression patterns between diploid and triploid gonads. *FoxG1*, *Cyp2J6*, *Fahp-MC*, *Sox11*, *Cyp4A2*, *Tra2A*, *FoxO*, *Far1*, *Sox5*, and *Mylip* were expressed at significantly higher levels in impaired triploid female (F-3nβ) gonads than in diploid female (F-2n) gonads, whereas their expression levels in developed triploid female gonads (F‑3nα) were intermediate, falling between the levels observed in F‑3nβ and F‑2n gonads. In contrast, *Uhrf1*, *RxrA1*, *Parpbp*, *Mettl9*, *Sox2*, *Jmjd8*, *Fem1B*, *Ccna2*, *Cdc25b*, *Bre1*, and *Mad2A* showed the opposite trend, with expression levels highest in F‑2n gonads, intermediate in F‑3nα gonads, and lowest in F‑3nβ gonads (Fig. 7A-I). Single-nucleus RNA‑seq analysis further revealed the cell type‑specific expression patterns of these genes. Specifically, *FoxG1* was predominantly expressed in vesicular connective tissue (VCT) cells (Fig. 7A). In addition, *Fahp-MC*, *Sox11*, and *Cyp4A2* were primarily localized to gonadal Niche cells (follicular cells) (Fig. 7C-E). *Cyp2J6* expression was detected in both VCT and Niche cells, while *Tra2A* exhibited a broad expression profile, encompassing VCT, Niche cells, and Germ cells (Fig. 7B, F). Additionally, *Uhrf1* expression was restricted to Germ cells of female gonads (Fig. 7G).

### Expression validation of mRNAs and ncRNAs by qRT‑PCR

**Fig. 8:**
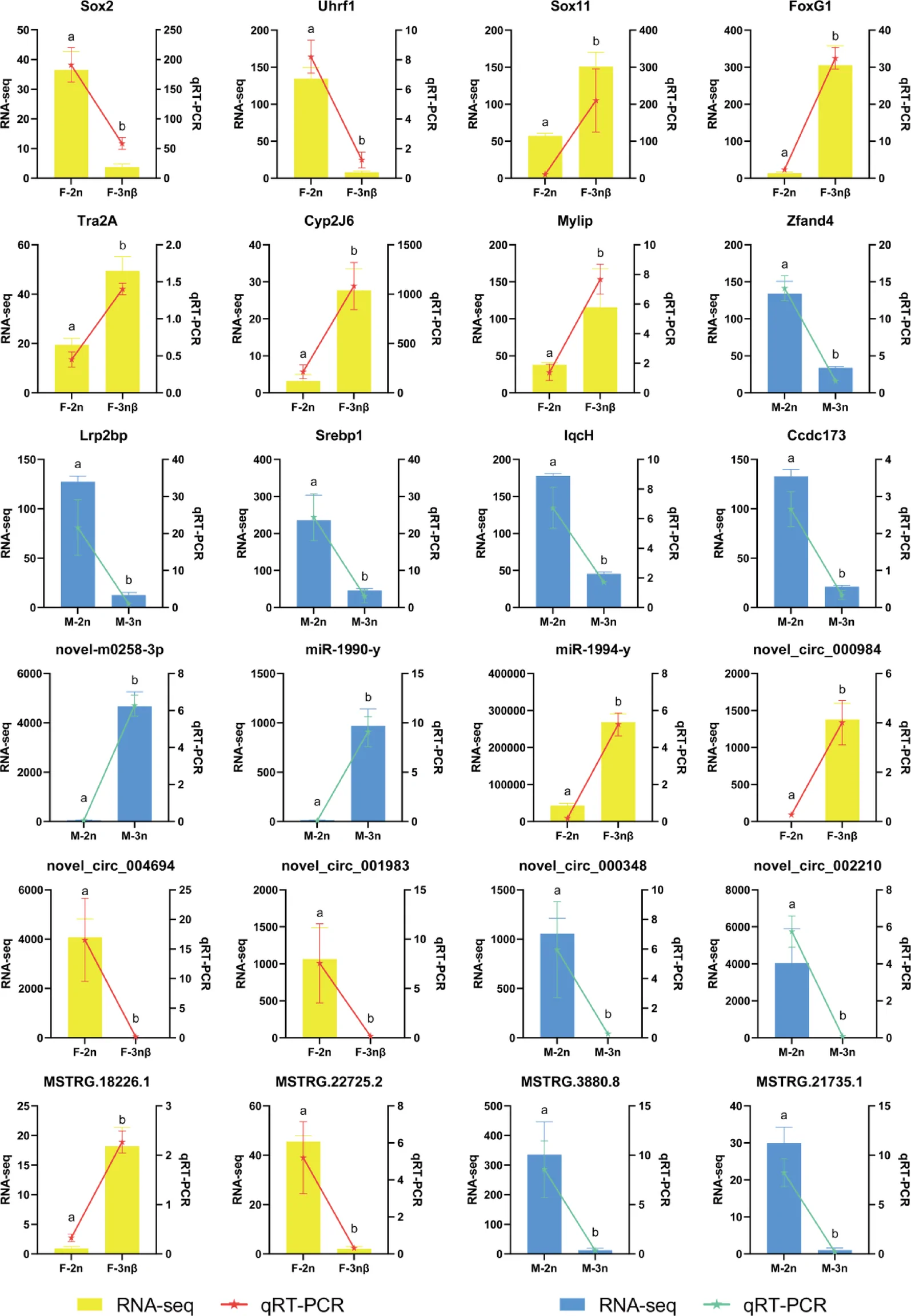
Expression validation of RNAs from the ceRNA network by qRT-PCR. Data are presented as mean ± SEM from three biological replicates and the statistical analysis was performed using the unpaired Student’s t-test. Significant differences between groups are indicated by different letters (*p* < 0.05)

In total, twelve mRNAs (*Sox2*, *Uhrf1*, *Sox11*, *FoxG1*, *Tra2A*, *Cyp2J6*, *Mylip*, *Zfand4*, *Lrp2bp*, *Srebp1*, *IqcH*, and *Ccdc173*), five circRNAs (novel_circ_000984, novel_circ_004694, novel_circ_001983, novel_circ_000348, and novel_circ_002210), four lncRNAs (MSTRG.18226.1, MSTRG.22725.2, MSTRG.3880.8, and MSTRG.21735.1), and three miRNAs (novel-m0258-3p, miR-1990-y, and miR-1994-y) were selected for qRT-PCR analysis to validate their expression patterns. Results of qRT-PCR for all selected DEGs, DECs, DELs, and DEMis were consistent with the RNA-seq data, thereby confirming the reliability and accuracy of the sequencing and the bioinformatic analyses results (Fig. 8).

## Discussion

Gametogenesis is a highly orchestrated event involving intricate cellular differentiation and precise molecular regulation [41]. Early research into the mechanisms of gametogenesis impairment primarily focused on identifying key genes, followed by functional validation and the dissection of their hierarchical regulatory relationships [7–14]. However, how key genes are embedded within higher-order post-transcriptional regulatory networks, particularly those mediated by non-coding RNAs, remains largely unresolved in molluscs. Triploid oysters, characterized by profound defects in gametogenesis, provide a unique system for dissecting the regulatory logic of gonadal development. Deciphering the molecular basis of gametogenesis impairment in triploid oysters offers a new perspective for elucidating the regulatory mechanisms of gametogenesis and gonadal development in molluscs.

In this study, we characterized the expression patterns of circRNAs in diploid and triploid gonads and constructed ceRNA networks from differentially expressed RNAs. Our integrative analyses demonstrate that circRNAs and ceRNA network components are broadly enriched in biological processes and signaling pathways fundamental to gametogenesis, highlighting a previously underappreciated regulatory layer regulating gonadal development in oysters. Furthermore, the identification of candidate genes within these networks and their spatiotemporal expression patterns provide mechanistic clues linking ceRNA dysregulation to impaired gametogenesis.

Numerous studies have demonstrated that ncRNAs regulate gametogenesis and gonadal development in various animals [18–29]. CircRNAs in particular, have emerged as key post-transcriptional regulators in both oogenesis or spermatogenesis [22–27]. In male oysters, source genes of DECs identified between diploid and triploid gonads were significantly enriched in pathways related to lipid and steroid metabolism and cell fate control. Downregulation of steroidogenic genes is well known to disrupt hormone biosynthesis and impair spermatogenesis [42]. Lipids serve as essential substrates for steroid biosynthesis, energy production, and membrane assembly during spermatogenesis, and their mobilization via adipocyte lipolysis is indispensable for male fertility [43–45]. *Ppar* genes are mediators of fatty acid metabolism in the testis and play critical roles in normal spermatogenesis [46]. Meanwhile, *p*53 plays a crucial role in regulating either cell proliferation or apoptosis in spermatogonia [47, 48]. Enrichment of circRNA source genes in these pathways and their aberrant expression in triploid males highlight the regulatory role of circRNAs in coordinating metabolic and cell‑cycle programs essential for molluscan spermatogenesis. In females, source genes of DECs identified between diploid and impaired triploid gonads were enriched in pathways relevant to carbohydrate and nucleotide metabolism. These findings point to a role for circRNAs in modulating genes involved in energy supply and nucleotide biosynthesis during oyster oogenesis, processes critical for sustained cell proliferation and oocyte growth. Consistent with this notion, *Hk1* and *Aldh1A1*—previously implicated in follicle development and meiotic initiation—are involved in modulating glycolysis, retinoic acid metabolism, and oogonial meiosis in vertebrates [49–52]. Collectively, these findings implicate circRNA‑mediated regulation of metabolic genes in meeting the energetic and biosynthetic demands of oogenesis in oysters.

CeRNA-mediated regulatory mechanism is evolutionarily conserved and has been implicated in governing gametogenesis and gonadal development across a broad spectrum of species, from mammals to arthropods [26–28]. In this study, we constructed an oogenesis‑related ceRNA network, and functional enrichment analysis revealed extensive involvement of metabolic pathways, mirroring the enrichment of DEC source genes and further supporting the critical role of metabolic regulation during oogenesis. In addition, multiple canonical signaling pathways—including Hedgehog, Wnt, Insulin, FoxO, AMPK, and PPAR—were enriched in the ceRNA network, indicating multilayered regulatory integration. These pathways are known regulators of folliculogenesis, oocyte maturation, and ovarian homeostasis in other animals [53–58]. Notably, previous study in triploid female oysters has demonstrated dysregulated AMPK/PPAR signaling in VCT cells, leading to a compromised oogenic nutrient reservoir [13]. The convergence of these pathways with the ceRNA network suggests that ceRNA‑mediated regulation may contribute to shaping the metabolic and signaling landscape essential for oogenesis.

Within the oogenesis‑related ceRNA network, we identified a subnetwork of candidate genes associated with oogenesis impairment. Several genes, including *FoxG1*, *Cyp2J6*, *Sox11*, *Tra2A*, were significantly upregulated in impaired triploid gonads. Single-nucleus RNA-seq analysis revealed that these genes were predominantly expressed in somatic gonadal cells. *FoxG1*, for instance, was dominantly expressed in VCT cells—a cell type containing numerous lipid droplets and glycogen particles, which serve to accumulate and provide nutrients during molluscan gametogenesis [59]. In vertebrates, *FoxG1* plays established roles in steroidogenesis and cell-cycle regulation during oogenesis [60, 61]. Similarly, *Sox11* and *Cyp2J* family genes have been linked to oocyte development in fish and mammals [62–64], while *Tra2A* is involved in oocyte-associated alternative splicing processes [65, 66]. The elevated expression of these genes in somatic cells of triploid female gonads suggests a dysregulated response that may interfere with germ cell development through ceRNA‑mediated mechanisms. On the contrary, *Uhrf1*, *Sox2*, *Ccna2*, *Cdc25b*, and *Bre1* showed high expression in diploid gonads but were markedly downregulated in impaired triploid oyster gonads, consistent with their conserved roles in promoting oogenesis [67–73]. *Uhrf1* is expressed in female germ cells and is essential for oocyte integrity and proliferation in multiple taxa [67, 68]. *Ccna2* and *Cdc25b* are key regulators of meiotic cell-cycle progression, while *Bre1* and *Sox2* functions in maintaining germ cell potency [69–73]. The coordinated suppression of these oogenesis-promoting genes within the ceRNA network provides a possible molecular explanation for the severe oogenic failure observed in triploid oysters.

A spermatogenesis‑associated ceRNA network was also constructed. While functional enrichment analysis showed no significant pathway enrichment, expression profiling indicated that candidate genes within this network were highly expressed in mature diploid male gonads, whereas their levels were markedly lower in triploid gonads. *Iqch*, *Ccdc173*, *Srebp1*, and *Gtpbp2* are implicated in sperm maturation, motility, and lipid homeostasis in vertebrates [74–77], together with other testis‑enriched genes such as *Lrp2bp* and *Gabarapl2*, further supporting the reproductive relevance of this ceRNA network [78, 79]. These findings collectively suggest that disrupted spermatogenesis in triploid males may involve ceRNA‑mediated suppression of genes critical for late‑stage spermatogenic differentiation and sperm function.

## Conclusion

In summary, this study provides the first comprehensive expression profile of circRNAs in diploid and triploid oyster gonads and constructs the associated ceRNA networks based on RNA interactions. Our results demonstrate that circRNA- and ceRNA-mediated regulatory circuits are potentially involved in coordinating metabolic, proliferative, and signaling pathways required for normal gametogenesis. Disruption of these networks in triploid oysters leads to aberrant regulation of conserved candidate genes, providing a mechanistic framework for understanding impaired oogenesis and spermatogenesis. These findings not only expand current knowledge of non-coding RNA function in molluscan reproduction, but also establish triploid oysters as a valuable system for investigating post-transcriptional regulation of gametogenesis in invertebrates.

### Perspectives and significance

In triploid oysters with impaired gametogenesis, energy is diverted from reproductive efforts to somatic growth, which confers the commercially valuable traits of sterility and fast growth. Consequently, cultivating sterile triploid oysters holds substantial economic and ecological value. Our findings highlight the potential roles of circRNAs and ceRNA networks in sex‑specific gametogenesis impairment in triploid oysters, with a focus on evolutionarily conserved candidate genes dysregulated through ceRNA mechanisms. These findings, however, are preliminary and require experimental validation. Future studies using dual‑luciferase reporter assays, CRISPR/Cas9, or gene overexpression will be needed to confirm the interactions and functions of key non‑coding ceRNAs and their target genes. These findings provide a new molecular perspective on triploid oyster sterility, thereby contributing to improved aquaculture practices and a broader understanding of reproduction in molluscs, with potential implications even for human reproductive biology.

## Supplementary Information

Supplementary Material 1.

## Data Availability

No datasets were generated or analysed during the current study.
